# PARP inhibitor Olaparib overcomes Sorafenib resistance through reshaping the pluripotent transcriptome in hepatocellular carcinoma

**DOI:** 10.1186/s12943-021-01315-9

**Published:** 2021-01-23

**Authors:** Xiao-Dong Yang, Fan-En Kong, Ling Qi, Jia-Xin Lin, Qian Yan, Jane Ho Chun Loong, Shao-Yan Xi, Yue Zhao, Yan Zhang, Yun-Fei Yuan, Ning-Fang Ma, Stephanie Ma, Xin-Yuan Guan, Ming Liu

**Affiliations:** 1 Department of Core Medical Laboratory, The Sixth Affiliated Hospital of Guangzhou Medical University, Qingyuan People’s Hospital, Guangzhou Medical University, Guangzhou, China; 2 Guangzhou Municipal and Guangdong Provincial Key Laboratory of Protein Modification and Degradation, School of Basic Medical Sciences, Guangzhou Medical University, Guangzhou, China; 3 Department of Clinical Oncology, Li Ka Shing Faculty of Medicine, State Key Laboratory of Liver Research, University of Hong Kong, Hong Kong, Hong Kong; 4 Guangdong Provincial People’s Hospital, Guangdong Academy of Medical Sciences, Guangzhou, China; 5 School of Biomedical Sciences, Li Ka Shing Faculty of Medicine, State Key Laboratory of Liver Research, University of Hong Kong, Hong Kong, Hong Kong; 6 State Key Laboratory of Oncology in Southern China, Collaborative Innovation Center for Cancer Medicine, Sun Yat-sen University Cancer Center, Guangzhou, China; 7 General, Visceral and Cancer Surgery, University Hospital of Cologne, Cologne, Germany; 8 Department of Pediatric Surgery, Guangzhou Institute of Pediatrics, Guangzhou Women and Children’s Medical Center, Guangzhou Medical University, Guangzhou, China; 9 Affiliated Cancer Hospital and Institute of Guangzhou Medical University, Guangzhou, 510095 China

**Keywords:** Pluripotent transcriptome, Embryonic stem cell, Sorafenib resistance, PARP inhibitors

## Abstract

Hepatocellular carcinoma (HCC) is one of the most common human malignancies worldwide with very poor prognosis. Resistance to targeted therapeutic drugs such as sorafenib remains one of the major challenges in clinical treatment. In the present study, PARP1 was found to be highly expressed in human embryonic stem cells, but progressively decreased upon specified hepatic differentiation. Reactivation of PARP1 expression was also detected in HCC residual tumors after sorafenib treatment in xenograft mouse model, indicating the potential important roles of PARP1 in stem cell pluripotency and HCC sorafenib treatment resistance. Overexpression of PARP1 was frequently observed in HCC patients, and closely associated with poor clinical outcome. Treatment of Sorafenib induced activation of DNA damage repair signaling, which is highly active and essential for maintenance of stem cell pluripotency in HCC residual tumors. PARP inhibitor Olaparib extensively suppressed the DNA damage repair signaling, and significantly inhibited the global pluripotent transcriptional network. The repression of key pluripotent transcriptional factors and DNA damage repair signaling by Olaparib was mainly through CHD1L-mediated condensation of the chromatin structure at their promotor regions. The global reshaping of the pluripotent transcriptome by Olaparib might reinforce Sorafenib in eliminating HCC residual tumors and enhance therapeutic efficiency.

**Supplementary Information:**

The online version contains supplementary material available at 10.1186/s12943-021-01315-9.

## Main text

Hepatocellular carcinoma is one of the most common human malignancies worldwide with poor prognosis [1]. Currently, the multi-kinase inhibitors Sorafenib was approved by FDA as first line treatment for unresectable advanced HCC. However, the benefit of the patients from the therapy is very limited, with a prolonged median overall survival rate less than 3 months [2, 3]. Thus, further investigation of the molecular mechanisms in drug resistance and development of novel therapeutic strategy is urgently needed.

Increasing evidences suggested that the hierarchy of cancer stem cells and their differentiated progenies contributed substantially to the heterogeneous tumor and therapeutic failure [4, 5]. To better understand the dynamic transcriptomic change during liver development, we have recently established a hepatocyte differentiation model, which specifically induced human embryonic stem cells (hESCs) to differentiate into mature hepatocytes along hepatic lineages [6]. Meanwhile, liver tumors were inoculated into immune deficient mice and treated with Sorafenib to establish a drug resistant model. Combining the transcriptomic data from the two model, PARP1 was identified to be the critical gene which actively expressed in embryonic stem cells and residual tumors after Sorafenib treatment, but progressively decreased along hepatic differentiation. PARP inhibitors have promising effects in inducing synthetic lethality in homozygous recombination deficient tumors in the clinic [7]. Our current study suggested that PARP1 was required for HCC tumor lineage plasticity and residual tumor survival potentially through CHD1L, a chromatin remodeling protein frequently amplified in HCC [8, 9]. Our study revealed a novel mechanism of PARP inhibitors in cancer treatment and further supported their extension to non-HR deficient tumors including HCC.

## PARP1 is activated in embryonic stem cells and the residual tumors after Sorafenib treatment

**Fig. 1:**
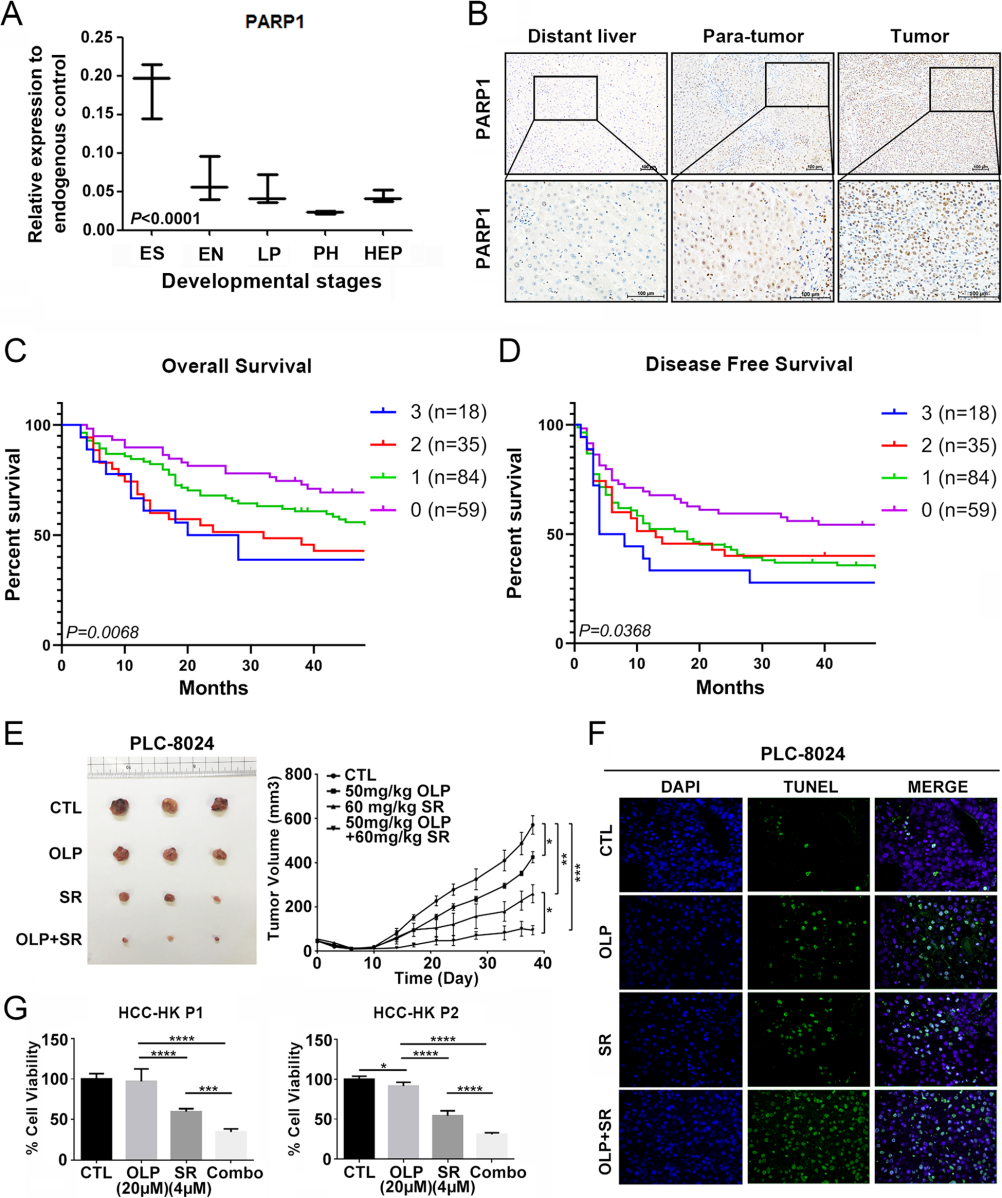
PARP1 is activated in embryonic stem cells and the residual tumors after Sorafenib treatment and significantly potentiated Sorafenib both in vitro and in vivo*.* **a** The relative expression of PARP1 normalized to 3 endogenous control (combination of GAPDH, ACTB, and RPL13A) at different developmental stages including embryonic stem cell (ES), endoderm (EN), liver progenitor cell (LP), and premature hepatocytes (PH) was shown in boxplot. One-way ANOVA test. *P* < 0.0001. **b** Representative immunohistochemical staining of PARP1 in clinical HCC tumor tissues and their adjacent liver tissues. Distant liver (liver tissues > 1 cm from the tumor margin); Para-tumor, (liver tissues < 1 cm from the tumor margin), Scale bar, 100 μm. **c** IHC staining of PARP1 was performed in a tissue microarray (TMA) containing 196 liver tumor tissues from HCC patients. Kaplan-Meier analysis indicated that the staining score of PARP1 was significantly correlated with poor overall survival (*P* = 0.0068) (**d**) and disease-free survival (*P* = 0.0368) of HCC patients. **e** PLC-8024 cells (2 × 10^6^ cells/mouse) were injected into nude mice at the right dorsal subcutaneously. The mice were randomly divided into four groups with different setup: treatment with PBS; Olaparib (50 mg/kg, thrice per week); Sorafenib (60 mg/kg, thrice per week) or both (*n* = 3). Tumor volumes were measured every three days. Data were shown as mean ± SD. **f** The apoptotic signals were detected by TUNEL staining of the tumor sections. Significant elevation of apoptosis was observed in tumors treated with both Sorafenib and Olaparib. **g** Tumor organoids derived from primary HCC patients (HCC-HK P1, HCC-HK P2) were treated with Olaparib (20 μM), Sorafenib (4 μM), or both, and their sensitivities to drug treatment were examined. *, *P* < 0.05, ***, *P* < 0.001, ****, *P* < 0.0001, independent t test

hESCs were differentiated into human hepatocytes along hepatic lineages. The whole differentiation process was defined with four stages, cells from the four different developmental stages were collected for transcriptomic profiling. (Additional file 1: Fig. S1A). Meanwhile, BALB/c nude mice were subcutaneous injected with PLC-8024 cells and treated with Sorafenib or vehicle control (Additional file 1: Fig. S1B, Additional file 2: file S1). Combining the two set of data together, PARP1 was identified to be significantly up-regulated both in embryonic stem cells and the residual tumors after Sorafenib treatment (Fig. 1a, Additional file 1: Fig. S1C). The expression of PARP1 remains low in distant normal liver tissues, but progressively increased from para-tumor liver tissues to the tumor tissues (Fig. 1b). High expression of PARP1 was detected in 53/196 (27%) HCC patients from a tissue microarray (Additional file 3: Tab S1, Additional file 1: Fig. S1D). Kaplan-Meier analysis indicated that the PARP1 staining score significantly stratified the overall survival (Fig. 1c) and disease-free survival (Fig. 1d) of HCC patients. Furthermore, univariate and multivariate cox regression analysis also proposed the high expression of PARP1 as an independent prognostic factor in HCC (Additional file 4: Tab S2).

## PARP inhibitor Olaparib inhibits tumorigenesis in HCC and significantly potentiates Sorafenib both in vitro and in vivo

PARP inhibitor Olaparib has already been approved by FDA in treatment of BRCA-mutated ovarian cancer and showed promising clinical benefit [10]. Functional assays proved that Olaparib could inhibit HCC cell growth and colony formation ability (Additional file 5: Fig. S2A, S2B). Sphere formation assay indicated a significant decrease in sphere number after Olaparib exposure (Additional file 5: Fig. S2C). A significant reduction of tumor size was also observed after exposes of Olaparib at the dose of 50 mg/kg (Additional file 5: Fig. S2D). Treatment of Olaparib alone or in combination with Sorafenib significantly inhibited cell colony formation in HCC cells with high pluripotency (Hep3B and Huh7) both under normal culture conditions or in the spheres which mimic stem cell microenvironment (Additional file 5: Fig. S2E, S2F, S2G). Similar results were found in cells treated with another PARP inhibitor Niraparib (Additional file 5: Fig. S2H, S2I). Olaparib significantly potentiated Sorafenib drug efficacy (Additional file 6: Fig. S3A and S3B). Western blot analysis further indicated that Sorafenib and Olaparib could induce apoptotic signaling (Additional file 6: Fig. S3C). A significant decrease in tumor size was observed when the mice were treated with Olaparib or Sorafenib alone, and enhanced tumor regression was observed when the mice were treated with both drugs (Fig. 1e, Additional file 6: Fig. S3D, and S3E). In situ TUNEL assay of the xenograft tumors confirmed the elevated level of apoptosis (Fig. 1f). Similar results were also found in HepG2 cells (Additional file 6: Fig. S3F, S3G, S3H, S3I, and S3J). Treatment of Olaparib also significantly potentiated the tumor inhibitory effects of Sorafenib in patient-derived HCC organoids (Fig. 1g).

## Olaparib extensively suppressed the DNA damage repair signaling potentially through CHD1L

**Fig. 2:**
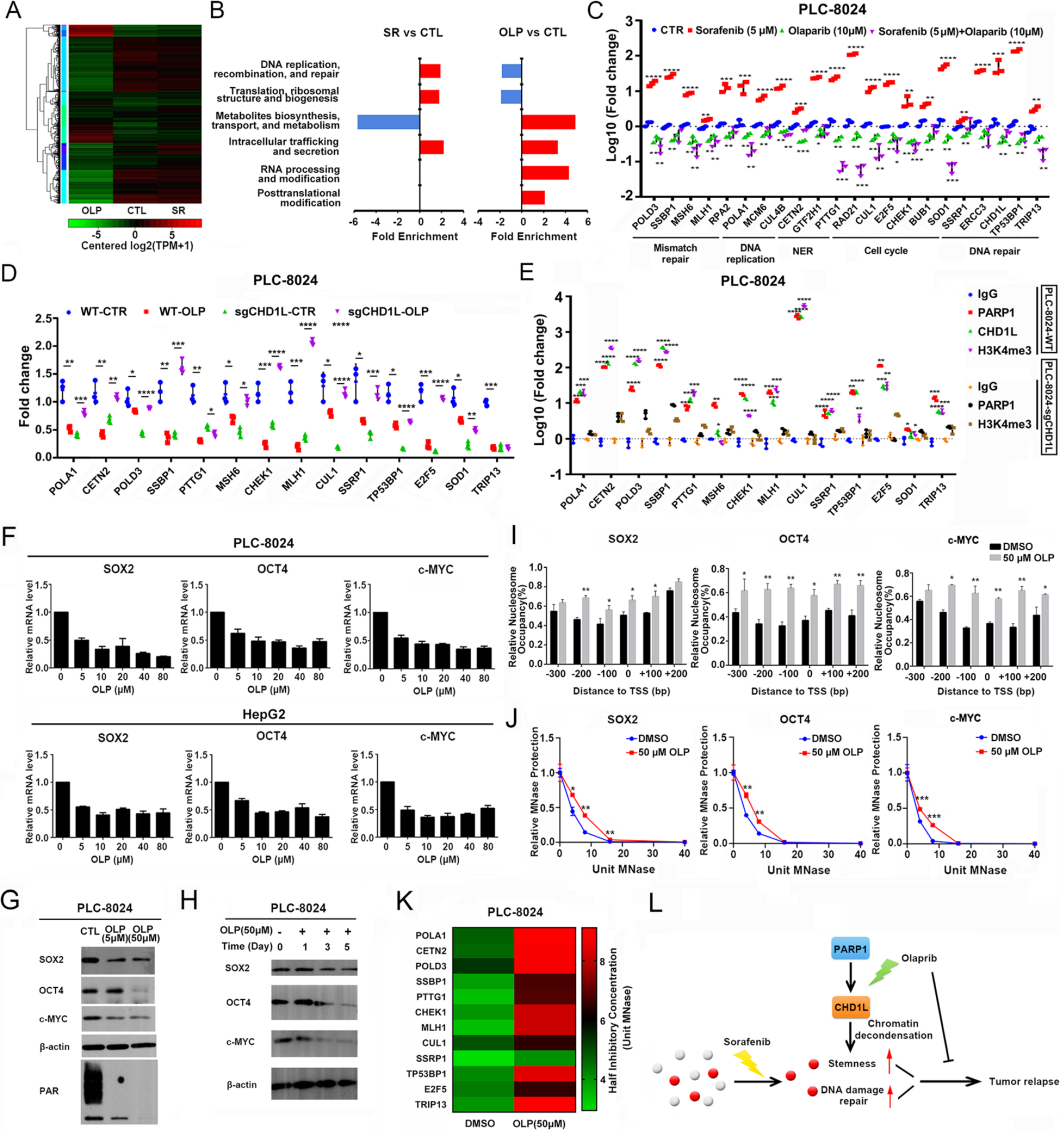
Olaparib extensively suppressed the DNA damage repair signaling and key pluripotent transcriptional factors potentially through chromatin remodeling protein CHD1L. **a** Hierarchical clustering analysis of residual tumors after treatment with Olaparib, Sorafenib, and vehicle control. **b** Gene ontology analysis of differentially expressed genes in different subgroups of residual tumors after drug treatment. **c** The representative DNA damage repair gene expression profiling in PLC-8024 cells treated with Olaparib, Sorafenib, combination of both and vehicle control was examined by qPCR, Mann-Whitney test. **d** Representative DNA damage repair gene expression profiling in wildtype PLC-8024 cells and CHD1L knock out PLC-8024 cells (8024-sgCHD1L) treated with Olaparib, Mann-Whitney test. **e** The binding of PARP1, CHD1L to the promoter regions of the representative DNA damage repair gene, as well as the active transcription histone marker H3K4me3 were examined by ChIP-qPCR both in wildtype PLC-8024 cells and 8024-sgCHD1L cells. Mann-Whitney test. **f** HCC cell lines PLC-8024 and HepG2 were treated with increasing doses of Olaparib, and the relative expression of key pluripotency transcriptional factors SOX2, OCT4, c-MYC were examined by qPCR. **g** PLC-8024 cells were treated with Olaparib at different concentrations (5 μM, 50 μM) and the relative expression of SOX2, OCT4, c-MYC were detected by western blot. The activity of PARP1 after Olaparib treatment was also examined by western blot. **h** SOX2, OCT4, c-MYC were detected by western blot at different time points after Olaparib treatment. **i** Genomic DNA extracted from PLC-8024 cells treated with or without Olaparib was further treated with 8 U MNase. The relative nucleosome occupancy at specific regions (− 300 bp to + 200 bp) nearby the transcription start sites (TSSs) of SOX2, OCT4, and c-MYC was detected by qPCR, independent t test. **j** Genomic DNA was treated with the indicated amount of MNase (0-40 U). The fraction of uncut DNA fragments at the TSSs of SOX2, OCT4, c-MYC and (**k**) DNA damage repair genes was determined by qPCR, independent t test. The half inhibitory MNase Unit was calculated and presented in the form of heatmap. (**l**) Model of Olaparib in potentiating Sorafenib in HCC treatment. *, *P* < 0.05, **, *P* < 0.01, ***, *P* < 0.001, ****, *P* < 0.0001

Transcriptome RNA sequencing was performed in residual tumors after treatment with Olaparib, Sorafenib, and vehicle control. Hierarchical clustering analysis revealed that the tumors treated with Olaparib showed the lowest global gene expression level and was segregated with other subgroups (Fig. 2a). Gene ontology analysis revealed that genes associated with DNA damage repair was up-regulated in HCC tumors treated with Sorafenib. Interestingly, absolute reverse effect was found in HCC tumors treated with Olaparib. This indicated that Olaparib might suppress the DNA damage repair signaling and counteract with the effects of Sorafenib on HCC tumors (Fig. 2b).

The DNA damage repair signaling molecules were significantly up-regulated after Sorafenib treatment but significantly suppressed by Olaparib (Fig. 2c). Similar results were found in Hep3B and Huh7 cells, as well as in PLC-8024 cells treated with another PARP inhibitor Niraparib (Additional file 7: Fig. S4A, S4B, S4C). CHD1L was dynamically expressed during hepatocyte differentiation (Additional file 7: Fig. S4D). Doxorubicin (Dox)-induced *CHD1L* knockout PLC-8024 cells were established using CRISPR/Cas9 gene editing methods (Additional file 7: Fig. S4E). The DNA damage repair genes were examined in wildtype PLC-8024 cells and cells with CHD1L knock out (sgCHD1L) treated with or without Olaparib. We found the suppression of DNA damage repair genes by Olaparib was totally abolished when CHD1L was deleted (Fig. 2d). Furthermore, both PARP1 and CHD1L was found to occupy the promoter regions of the DNA damage repair genes and associated with open chromatin histone methylation marker H3K4me3. Deletion of CHD1L abolished the binding of PARP1 to the promoters of DNA damage repair genes (Fig. 2e). These findings suggested that PARP1 might form a complex with CHD1L and maintain an “open chromatin” status at the promoter regions of critical DNA damage repair genes.

## The DNA damage repair signaling suppressed by Olaparib might be critical in stem cell pluripotency and Sorafenib resistance

The residual tumors after Sorafenib might be eliminated with treatment of Olaparib (Additional file 7: Fig. S4F). Genes up-regulated after Sorafenib treatment, but suppressed by Olaparib showed high expression in hESCs but significantly decreased in the differentiated hepatocytes, which was in accordance with the expression pattern of DNA damage repair genes and key pluripotent transcriptional factors (Additional file 7: Fig. S4G, Fig. S4H, and S4I). This indicated that embryonic stem cells might rely on active DNA damage repair to maintain pluripotency, and they might contribute substantially to Sorafenib resistance in HCC treatment. Deletion of CHD1L also decreased the binding of PARP1 and active histone marker H3K4me3 at promoters of pluripotent transcriptional factors (Additional file 7: Fig. S4J). A dose-dependent decrease of pluripotent transcriptional factors was observed after Olaparib treatment (Fig. 2f). The inhibitory effects were further confirmed at protein level in a dose- and time- dependent manner (Fig. 2g, h and Additional file 8: Fig. S5A). Immunohistochemical staining (IHC) also confirmed the inhibition of pluripotency transcriptional factors in xenograft tumors after Olaparib treatment (Additional file 8: Fig. S5B).

## Olaparib might repress the key pluripotent transcriptional factors and DNA damage repair genes through condensation of chromatin structure

The open chromatin architecture could present more nucleosome-free promoter region, which was in turn readily digested by micrococcal nuclease (MNase). Inhibition of PARP1 by Olaparib could reverse such process, and thus exhibited a “protection” effect, leading to reduced digestion and consequently more uncut DNA retained for qPCR detection. After MNase treatment, the number of uncut DNA fragments near the promoter region of target genes were determined by qPCR in cells with or without Olaparib exposure (Fig. 2i). The tumor cells were also treated with different concentrations of MNase to monitor the dynamic chromatin structure change and sensitivity to MNase digestion. The results indicated that PARP1 could sustain the open chromatin structure for SOX2, OCT4, c-MYC. Pharmaceutical interruption of PARP1 by Olaparib could reverse the process and might account for its role in suppressing the DNA damage repair genes, as well as the whole HCC pluripotent transcriptome (Fig. 2j, k, Additional file 8: Fig. S5C).

In conclusion, we found Sorafenib treatment could retain resistant tumor cells characterized with elevated cancer stemness and activation of DNA damage repair signaling. PARP1, which is highly activated in embryonic stem cells and Sorafenib resistant cancer cells, might be responsible for the active transcription of the pluripotent transcriptional factors and DNA damage repair signaling through maintaining an “open chromatin” structure. PARP inhibitor Olaparib extensively suppressed the pluripotent transcriptome through condensation of the chromatin structure and might greatly reinforce Sorafenib in eliminating HCC further in the clinic (Fig. 2l).

## Supplementary Information

**Additional file 1: Figure S1.** Identification of PARP1 as a potential therapeutic target in HCC.

**Additional file 2.**

**Additional file 3: Table S1.**

**Additional file 4: Table S2.**

**Additional file 5: Figure S2.** PARP inhibitor Olaparib inhibits tumorigenesis in HCC

**Additional file 6: Figure S3.** Olaparib significantly potentiated Sorafenib both in vitro and in vivo

**Additional file 7: Figure S4.** Olaparib extensively suppressed the DNA damage repair signaling potentially through chromatin remodeling protein CHD1L

**Additional file 8: Figure S5.** Olaparib might repress the key pluripotency transcriptional factors through condensation of chromatin structure

**Additional file 9: Table S3.**

## Data Availability

All data and material have been provided in the Additional File: documents.

## Abbreviations

hESCs: Human embryonic stem cells
PARP1: Poly (ADP-ribose) polymerase 1
CHD1L: Chromodomain helicase/ATPase DNA binding protein1-like
HCC: Hepatocellular carcinoma
ES: Embryonic stem cell
EN: Endoderm
LP: Liver progenitor cell
PH: Premature hepatocytes
HR: Homologous recombination
TSS: Transcriptional star site
qPCR: Quantitative real time PCR
