# Supplementary figures and images for: PARP inhibitor Olaparib overcomes Sorafenib resistance through reshaping the pluripotent transcriptome in hepatocellular carcinoma
Source: Mol Cancer. 2021 Jan 23;20:20. doi: 10.1186/s12943-021-01315-9 (PMC7824946; doi:10.1186/s12943-021-01315-9)

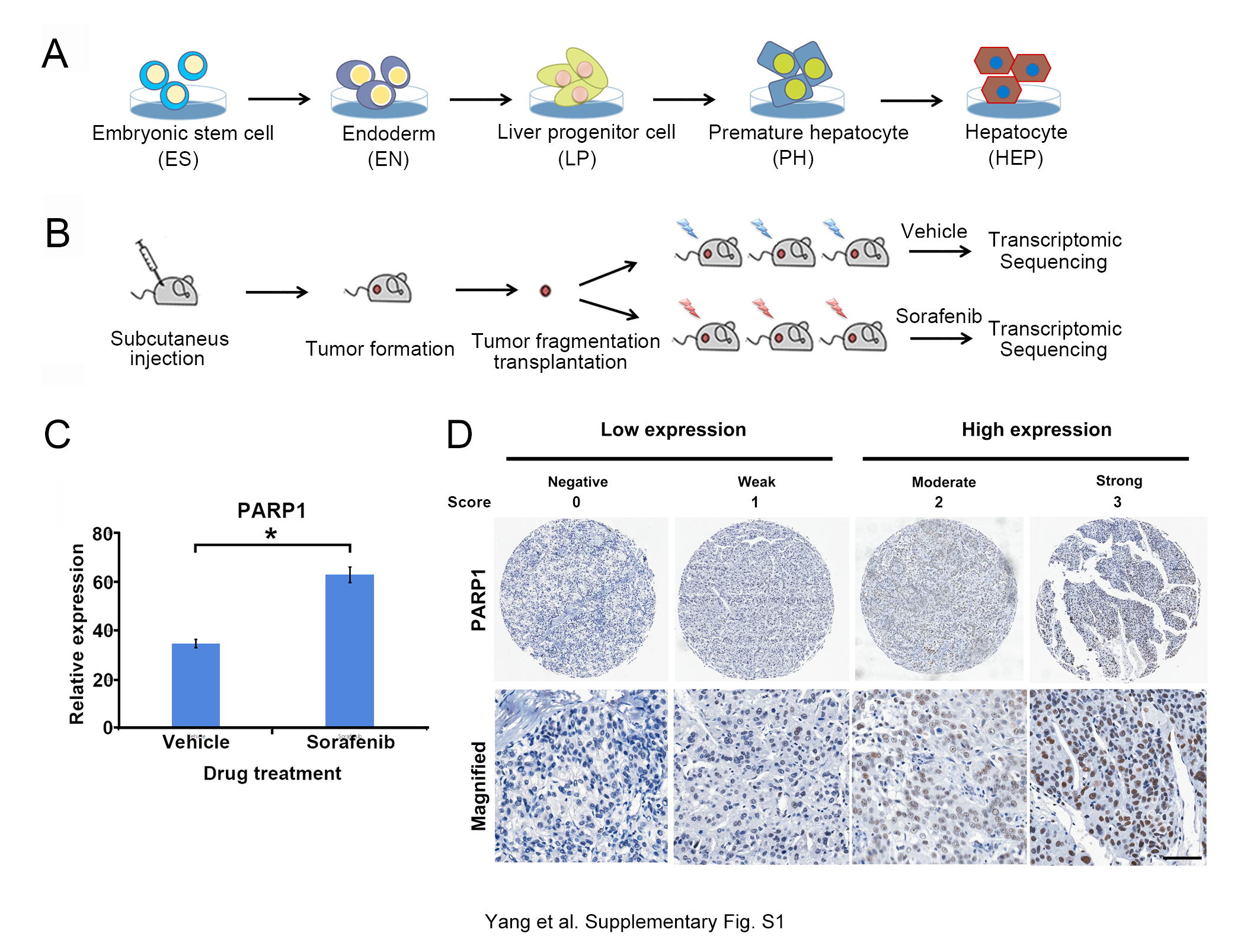

Supplement: Supplementary file 1 — Additional file 1: Figure S1. Identification of PARP1 as a potential therapeutic target in HCC. [file 12943_2021_1315_MOESM1_ESM.tiff]

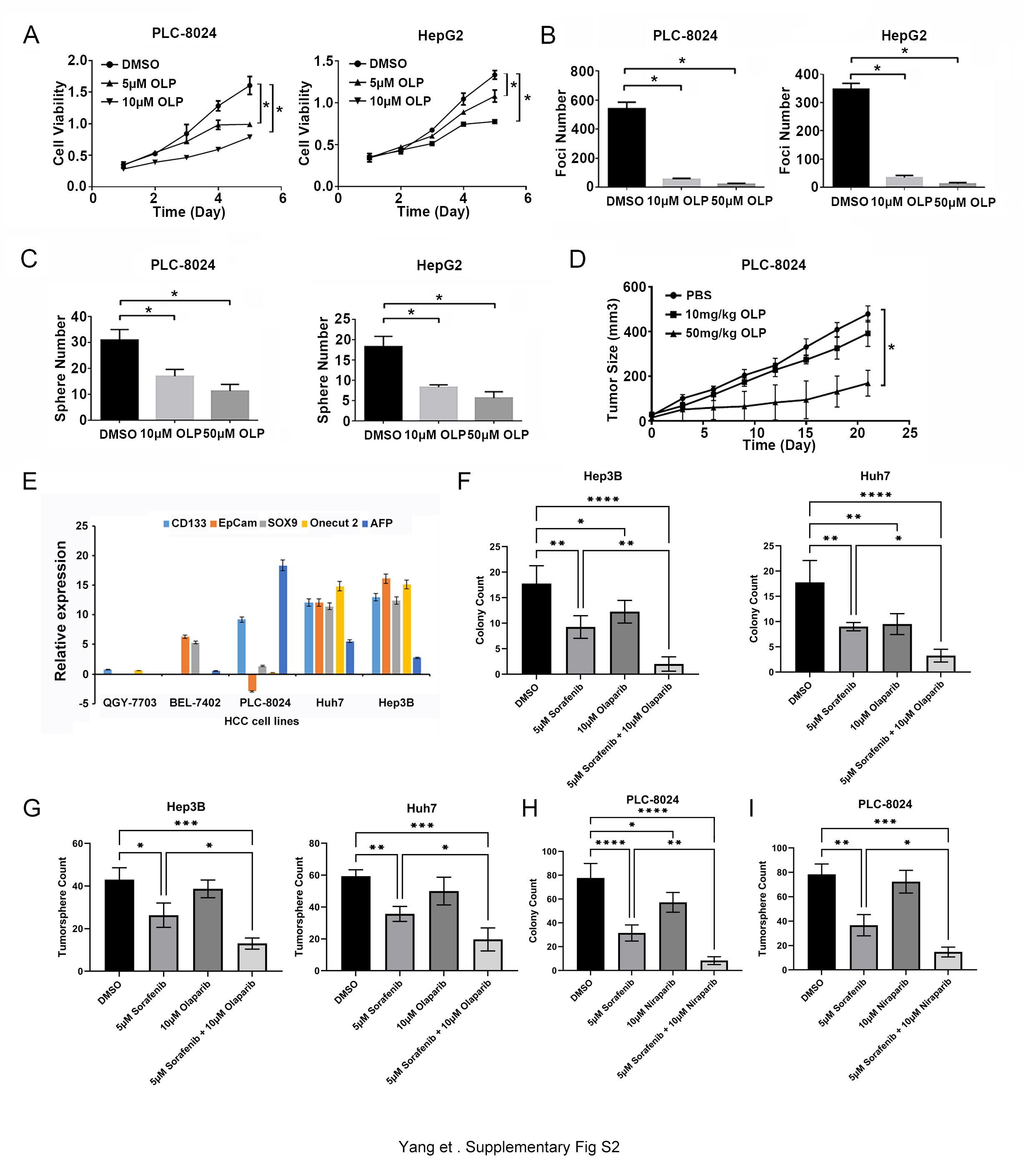

Supplement: Supplementary file 5 — Additional file 5: Figure S2. PARP inhibitor Olaparib inhibits tumorigenesis in HCC [file 12943_2021_1315_MOESM5_ESM.tif]

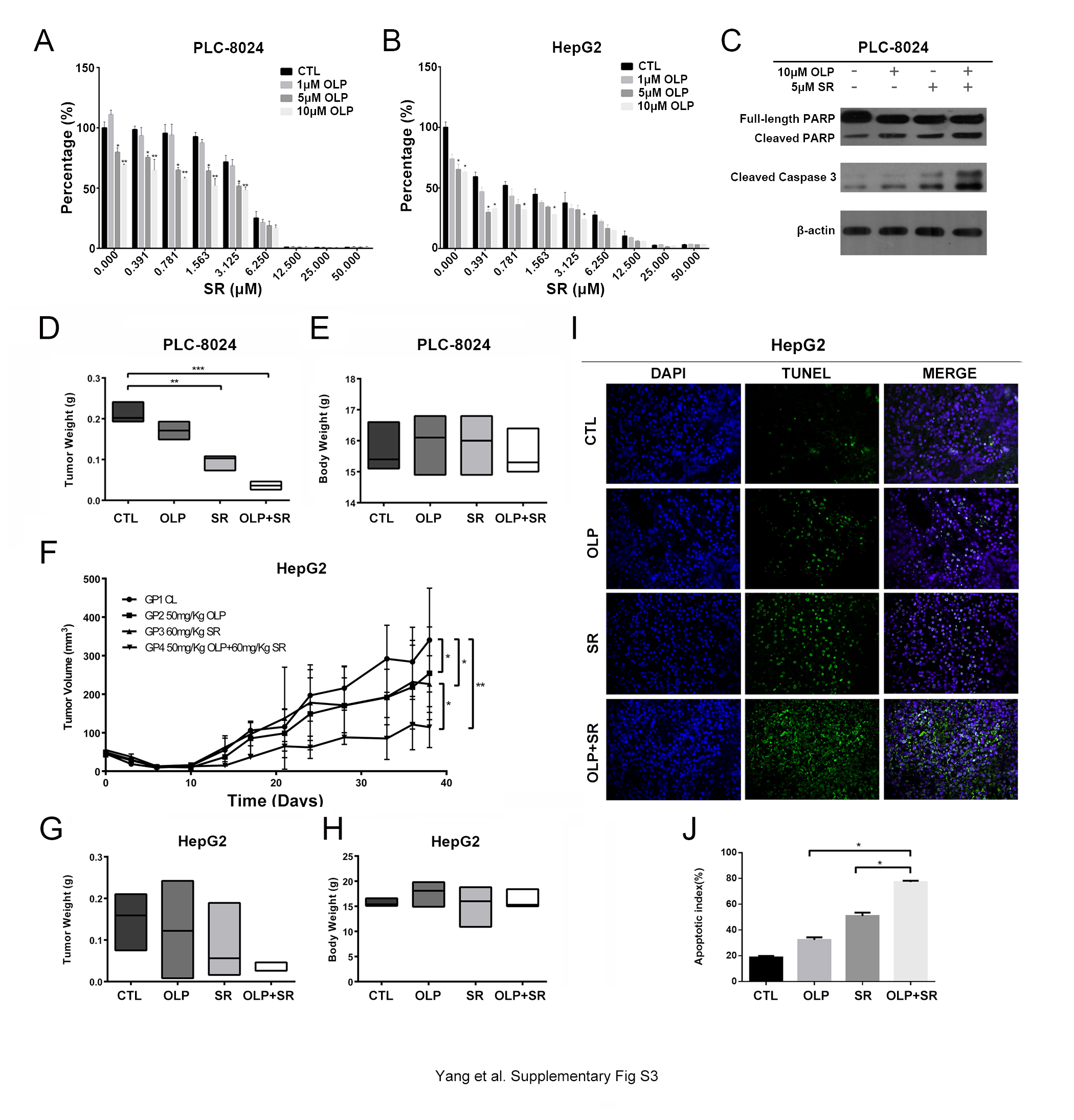

Supplement: Supplementary file 6 — Additional file 6: Figure S3. Olaparib significantly potentiated Sorafenib both in vitro and in vivo [file 12943_2021_1315_MOESM6_ESM.tif]

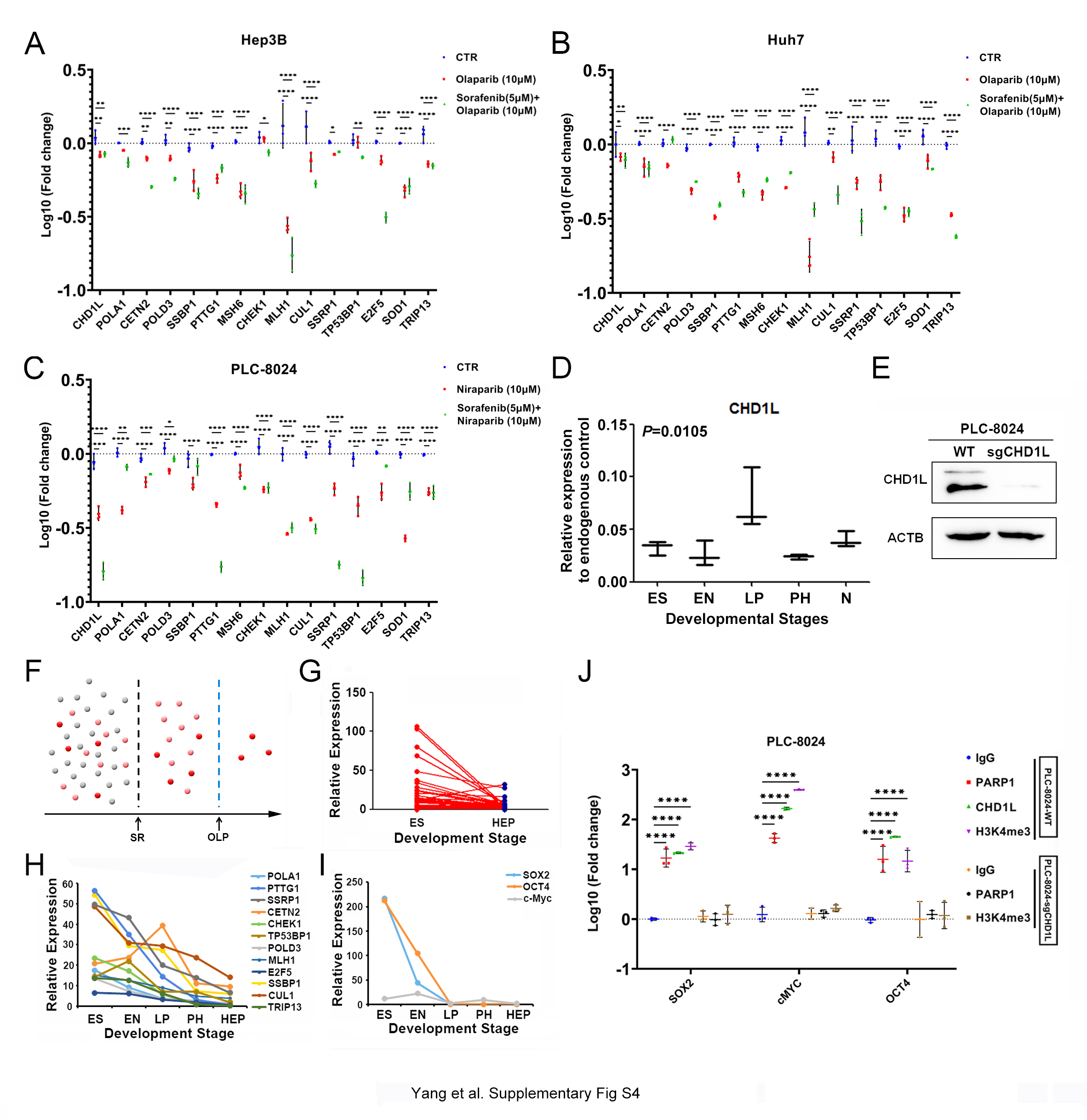

Supplement: Supplementary file 7 — Additional file 7: Figure S4. Olaparib extensively suppressed the DNA damage repair signaling potentially through chromatin remodeling protein CHD1L [file 12943_2021_1315_MOESM7_ESM.tif]

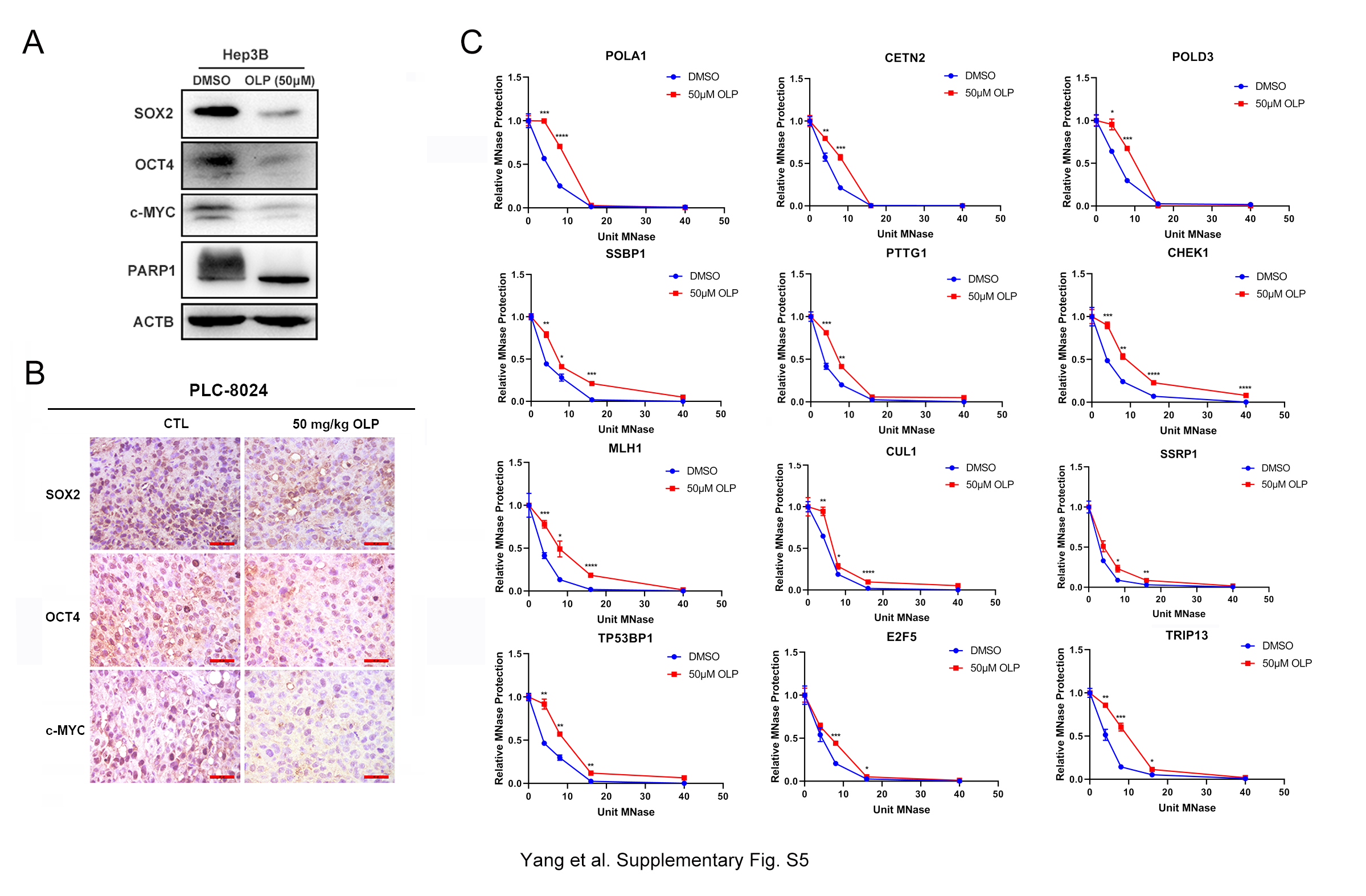

Supplement: Supplementary file 8 — Additional file 8: Figure S5. Olaparib might repress the key pluripotency transcriptional factors through condensation of chromatin structure [file 12943_2021_1315_MOESM8_ESM.tif]
