# Supplementary material for: PARP inhibitor Olaparib overcomes Sorafenib resistance through reshaping the pluripotent transcriptome in hepatocellular carcinoma
Source: Mol Cancer. 2021 Jan 23;20:20. doi: 10.1186/s12943-021-01315-9 (PMC7824946; doi:10.1186/s12943-021-01315-9)
Supplement: Supplementary file 3 — Additional file 3: Table S1. [file 12943_2021_1315_MOESM3_ESM.docx]

**Table S1.** Clinical characteristics of the HCC patients in tissue array

| **Clinical Features** | **High PARP1 group** | **Low PARP1 group** | **P value** |
| --- | --- | --- | --- |
| Age, (year), No. (%) |  |  |  |
| ≤50 | 34 (17.3) | 73 (37.2) | 0.102 |
| >50 | 19 (9.7) | 70 (35.7) |  |
| Gender, No. (%) |  |  |  |
| Male | 44 (22.4) | 122 (62.2) | 0.692 |
| Female | 9 (4.6) | 21 (10.7) |  |
| Tumor Stage, No. (%) |  |  |  |
| I | 31 (15.8) | 86 (43.9) | 0.964 |
| II | 7 (3.6) | 17 (8.7) |  |
| III | 15 (7.7) | 40 (20.4) |  |
| HBV status, No. (%) |  |  |  |
| HBV- | 5 (2.6) | 26 (13.3) | 0.136 |
| HBV+ | 48 (24.5) | 117 (59.7) |  |
| ALT, (U/L), No. (%) |  |  |  |
| ≤40 | 23 (11.7) | 78 (39.8) | 0.165 |
| >40 | 30 (15.3) | 65 (33.2) |  |
| AST, (U/L), No. (%) |  |  |  |
| ≤40 | 26 (13.3) | 90 (45.9) | 0.079 |
| >40 | 27 (13.8) | 53 (27.0) |  |
| AFP, (ng/ml), No. (%) |  |  |  |
| ≤400 | 27 (13.8) | 80 (40.8) | 0.532 |
| >400 | 26 (13.3) | 63 (32.1) |  |
| Multiple tumor, No. (%) |  |  |  |
| - | 44 (22.4) | 109 (55.6) | 0.307 |
| + | 9 (4.6) | 34 (17.3) |  |
| Tumor embolus, No. (%) |  |  |  |
| - | 41 (20.9) | 119 (60.7) | 0.347 |
| + | 12 (6.1) | 24 (12.2) |  |
| Tumor diameter, (cm), No. (%) |  |  |  |
| ≤5 | 21 (10.7) | 54 (27.6) | 0.812 |
| >5 | 32 (16.3) | 89 (45.4) |  |
