# Supplementary material for: PARP inhibitor Olaparib overcomes Sorafenib resistance through reshaping the pluripotent transcriptome in hepatocellular carcinoma
Source: Mol Cancer. 2021 Jan 23;20:20. doi: 10.1186/s12943-021-01315-9 (PMC7824946; doi:10.1186/s12943-021-01315-9)
Supplement: Supplementary file 4 — Additional file 4: Table S2. [file 12943_2021_1315_MOESM4_ESM.docx]

**Table S2.** Univariate and multivariate Cox regression analysis of DFS of HCC patients in tissue array

|  | **Univariate Analysis** | | | **Multivariate Analysis** | | |
| --- | --- | --- | --- | --- | --- | --- |
|  | HR | 95% CI | P value | HR | 95% CI | P value |
| **Age (year)** |  |  |  |  |  |  |
| >50 vs. ≤50 | 1.406 | 0.953-2.075 | 0.086 | NA | NA | NA |
| **Gender** |  |  |  |  |  |  |
| Male vs. Female | 0.615 | 0.380-0.994 | **0.047** | 0.576 | 0354-0.938 | **0.027** |
| **Tumor Stage** |  |  |  |  |  |  |
| III vs. I/II | 3.183 | 2.139-4.737 | **0.000** | 2.640 | 1.570-4.438 | **0.000** |
| **HBV** |  |  |  |  |  |  |
| HBV+ vs. HBV- | 1.054 | 0.609-1.824 | 0.851 | NA | NA | NA |
| **ALT (U/L)** |  |  |  |  |  |  |
| >40 vs. ≤40 | 1.321 | 0.895-1.950 | 0.162 | NA | NA | NA |
| **AST (U/L)** |  |  |  |  |  |  |
| >40 vs. ≤40 | 2.101 | 1.422-3.103 | **0.000** | 1.662 | 1.105-2.501 | **0.015** |
| **AFP (ng/ml)** |  |  |  |  |  |  |
| >400 vs. ≤400 | 1.775 | 1.201-2.622 | **0.004** | 1.532 | 1.011-2.323 | **0.044** |
| **Multiple tumor** |  |  |  |  |  |  |
| + vs. - | 1.909 | 1.238-2.942 | **0.003** | 1.082 | 0.624-1.875 | 0.780 |
| **Tumor embolus** |  |  |  |  |  |  |
| + vs. - | 2.154 | 1.378-3.367 | **0.001** | 1.478 | 0.918-2.378 | 0.108 |
| **Tumor diameter (cm)** |  |  |  |  |  |  |
| >5 vs. ≤5 | 2.036 | 1.322-3.136 | **0.001** | 1.493 | 0.939-2.373 | 0.090 |
| **PARP1 expression** |  |  |  |  |  |  |
| High vs. Low | 1.639 | 1.082-2.484 | **0.020** | 1.639 | 1.069-2.515 | **0.024** |
