# Supplementary material for: PARP inhibitor Olaparib overcomes Sorafenib resistance through reshaping the pluripotent transcriptome in hepatocellular carcinoma
Source: Mol Cancer. 2021 Jan 23;20:20. doi: 10.1186/s12943-021-01315-9 (PMC7824946; doi:10.1186/s12943-021-01315-9)
Supplement: Supplementary file 9 — Additional file 9: Table S3. [file 12943_2021_1315_MOESM9_ESM.docx]

**Table S3.** Sequences of primers used in qPCR.

| Primer | Seuence (5’-3’) |
| --- | --- |
| RT-POLA1-F | AGAAGCTCGCAGTGACAAAAC |
| RT-POLA1-R | AGGTGGTGGAGTTATTTGAGGT |
| RT-CETN2-F | GCATCAAGTTCTCAGCGAAAAAG |
| RT-CETN2-R | CCATCCGCATCGAAAAGATCAA |
| RT-CUL4B-F | CAAACGGCCTAGCCAAATCTT |
| RT-CUL4B-R | CAGTTTTTGCCAGGTTTCATCTG |
| RT-POLD3-F | ACCAACAAGGAAACGAAAACAGA |
| RT-POLD3-R | GGTTCCGTGACAGACACTGTA |
| RT-SSBP1-F | TGAGTCCGAAACAACTACCAGT |
| RT-SSBP1-R | CCTGATCGCCACATCTCATTAG |
| RT-PTTG1-F | ACCCGTGTGGTTGCTAAGG |
| RT-PTTG1-R | ACGTGGTGTTGAAACTTGAGAT |
| RT-MSH6-F | TCATCCGCGAGAAAGGGAAAT |
| RT-MSH6-R | ATCTGCACGTTGCATTGCTCT |
| RT-CHEK1-F | ACTTACTGCAATGCTCGCTGG |
| RT-CHEK1-R | TTGAGGGGTTTGTTGTACCATC |
| RT-RAD21-F | GGATAAGAAGCTAACCAAAGCCC |
| RT-RAD21-R | CTCCCAGTAAGAGATGTCCTGAT |
| RT-BUB1-F | ACAATCAACGGAGAAAGCATGA |
| RT-BUB1-R | CTCCACCACCTGATGCAACT |
| RT-MLH1-F | CAACAAGTCTGACCTCGTCTTC |
| RT-MLH1-R | CCGGGAATCTGTACGAACCAT |
| RT-GTF2H1-F | GACCTTGTTGTGAGTCAAGTGA |
| RT-GTF2H1-R | CCTGCTTATGATTGGATGTGGAA |
| RT-CUL1-F | GGTTCGCCGTGAATGTGAC |
| RT-CUL1-R | CCCCAATTCCACGTAAGACTGT |
| RT-SSRP1-F | TGACTACAAGATCCCCTACACC |
| RT-SSRP1-R | GAGTTTGGCCTTGCTTGATTG |
| RT-MCM6-F | ACCTGCCTACCAGACACAAGA |
| RT-MCM6-R | ACAGAAAAGTTCCGCTCACAAG |
| RT-TP53BP1-F | CTCCAGACGCACAAAGAAAATCC |
| RT-TP53BP1-R | ACCTGACTGATGGAACCACAT |
| RT-ERCC3-F | ACCCCGTGATCCGAGAATG |
| RT-ERCC3-R | TGCTTGTGAAAGTCTCTGTGATG |
| RT-E2F5-F | TGGCAACTCAAAATCTGCCTG |
| RT-E2F5-R | TTGTAGTCATCTGCCGGGGTA |
| RT-SOD1-F | GGTGGGCCAAAGGATGAAGAG |
| RT-SOD1-R | CCACAAGCCAAACGACTTCC |
| RT-RPA2-F | CACAGGTCACTATTGTGGGGA |
| RT-RPA2-R | GCATGATCTTAAAGGCTACCAGG |
| RT-TRIP13-F | ACTGTTGCACTTCACATTTTCCA |
| RT-TRIP13-R | TCGAGGAGATGGGATTTGACT |
| POLA1/0-F | CCTCCCGAGCCGCTGATT |
| POLA1/0-R | TGCCATGGTCCCGAATCTCC |
| CETN2/0-F | GGCCCTGCACCAATAGGGA |
| CETN2/0-R | ACAGAGCGGCAGCACTCAC |
| POLD3/0-F | GAGGGCCGCCGTAGTGC |
| POLD3/0-R | ACCCGCCTCTAACCCCT |
| SSBP1/0-F | GAACTTCAGAGTTAAGGGGAGCCG |
| SSBP1/0-R | GACGATCTAACCCGAGCAGCC |
| PTTG1/0-F | AATTGGGCCGCGAGTTGTG |
| PTTG1/0-R | AACAGCCGCATTCATCTGAGG |
| MSH6/0-F | CAGTGTGCCAGCCCCG |
| MSH6/0-R | CGCCGGAGGAACCCG |
| CHEK1/0-F | GGACACACCACCAAGCCC |
| CHEK1/0-R | AGTGGCTCCTGCCTGTAATTC |
| MLH1/0-F | AGGAAGAGCGGACAGCGA |
| MLH1/0-R | CCACCCTTCAGCGGCAG |
| CUL1/0-F | CTCATCACATCTTTTGCCCAGATGA |
| CUL1/0-R | TTCAACCTAAATACTACCCGCTGCT |
| SSRP1/0-F | GTCTGACCAGAGCAGTAAACACCAC |
| SSRP1/0-R | GGGAACTGACTTTGGAAACCATGTC |
| TP53BP1/0-F | CTGGCTGAAAACAACCAAGAACTACTG |
| TP53BP1/0-R | ATTGTGAGCTCCTTAAGGGCAGAG |
| E2F5/0-F | GTGTTTCTAGGCAACGGCGG |
| E2F5/0-R | CCGGGGAAGCGTGAATGA |
| SOD1/0-F | TGCGAGGCGATTGGTTTGG |
| SOD1/0-R | TCGCCCTTCAGCACGCAC |
| TRIP13/0-F | TTCCGGGTCAGGAGGTGGT |
| TRIP13/0-R | CACCTCACGCCCAGCG |
| Sox2/0-F | CGCTAGAAACCCATTTATTCCC |
| Sox2/0-R | GCCTTGACAACTCCTGATACTT |
| Sox2/-300-F | CATTTAAGTACCCTGCACCAAA |
| Sox2/-300-R | TCGAAGGAAGTGGGTAAACAG |
| Sox2/+100-F | AGTAGTTTGCTGCCTCTTTAAG |
| Sox2/+100-R | ACTTCTCTCCCTTTCTTTCTCT |
| Sox2/+200-F | AATAACAATCATCGGCGGCGG |
| Sox2/+200-R | GCGCTTCCCTCCTCCTCT |
| Sox2/-200-F | GCTGCGAGAGGGGATACAAA |
| Sox2/-200-R | GAGGAGGGGGAGGAGGC |
| Sox2/-100-F | GGCCCCGCCCCCTTT |
| Sox2/-100-R | CTCCTCCACTCGAGCCCA |
| Oct4/0-F | AGAGGTCAAGGCTAGTGGGT |
| Oct4/0-R | GCTTGCGAAGGGACTACTCA |
| Oct4/+100-F | CCATGGCGGGACACCTG |
| Oct4/+100-R | TCACCTCCACCACCTGGA |
| Oct4/+200-F | TGATCCTCGGACCTGGCTAA |
| Oct4/+200-R | CTCAGAGCCTGGCCCAAC |
| Oct4/-100-F | CTTCCACAGACACCATTGCCA |
| Oct4/-100-R | AGAAACTGAGGCGAAGGATG |
| Oct4/-200-F | AGTCTGGGCAACAAAGTGA |
| Oct4/-200-R | CCCTCCAGGACCTCAGTG |
| Oct4/-300-F | CCACTTAGGAGGCTGGAG |
| Oct4/-300-R | GGAGTACAATGATGGCTCAA |
| MYC/0-F | ATAATGCGAGGGTCTGGACG |
| MYC/0-R | GCCGCGAGCAGCACA |
| MYC/+100-F | GAAGGGCAGGGCTTCTCAG |
| MYC/+100-R | CGATCCCTCCCTCCGTTCTT |
| MYC/+200-F | AATTCCAGCGAGAGGCAGAG |
| MYC/+200-R | CCCGGCTCTTCCACCCTA |
| MYC/-100-F | CCCACCCTCCCCATAAGCG |
| MYC/-100-R | TTTTCTTTTCCCCCACGCCCT |
| MYC/-200-F | GGTAGGCGCGCGTAGTTAAT |
| MYC/-200-R | GAGCACTCTAGCTCTAGGATGTA |
| MYC/-300-F | GTTCCGCCTGCGATGATTT |
| MYC/-300-R | TTTCTCTGCTGCTCCTCCGTA |
| RT-PARP1-F | CGGAGTCTTCGGATAAGCTCT |
| RT-PARP1-R | TTTCCATCAAACATGGGCGAC |
| RT-OCT4-F | CAAAGCAGAAACCCTCGTGC |
| RT-OCT4-R | TCTCACTCGGTTCTCGATACTG |
| RT-SOX2-F | TGGACAGTTACGCGCACAT |
| RT-SOX2-R | CGAGTAGGACATGCTGTAGGT |
| RT-cMYC-F | GGCTCCTGGCAAAAGGTCA |
| RT-cMYC-R | CTGCGTAGTTGTGCTGATGT |
| RT-CHD1L-F | GGTGGAGTTGGCATGAACTT |
| RT-CHD1L-R | CACTCAACTGGAGGTCAGCA |
